# Global “worming”: Climate change and its projected general impact on human helminth infections

**DOI:** 10.1371/journal.pntd.0006370

**Published:** 2018-07-19

**Authors:** Alexander J. Blum, Peter J. Hotez

**Affiliations:** 1 Department of Surgery, Baylor College of Medicine, Houston, Texas, United States of America; 2 Texas Children's Hospital Center for Vaccine Development, Houston, Texas, United States of America; 3 National School of Tropical Medicine, Baylor College of Medicine, Houston, Texas, United States of America; 4 Department of Pediatrics, Baylor College of Medicine, Houston, Texas, United States of America; 5 Department of Molecular Virology and Microbiology, Baylor College of Medicine, Houston, Texas, United States of America; 6 Department of Biology, Baylor University, Waco, Texas, United States America; 7 James A. Baker III Institute of Public Policy, Rice University, Houston, Texas, United States America; 8 Scowcroft Institute of International Affairs, Bush School of Government and Public Policy, Texas A&M University, College Station, Texas, United States of America; University of Florida, UNITED STATES

Projected climate changes in the coming decades are expected to affect the prevalence and incidence of some human helminth infections.

Nearly one-fifth of the way through the 21st century, humanity is confronted by the realities of a quickly changing climate: warmer temperatures, alterations in rainfall patterns and distributions, floods and droughts, and other extreme weather events. All of these changes are expected to intensify in the coming decades. In concert with other global trends relevant to populations affected by neglected tropical diseases (NTDs), including social determinants such as urbanization, conflict, human migrations, and economic shifts [1,2], climate change will play a significant role in determining the future viability of helminth species and the emergence or decline of human helminthiases. Still another factor will be ongoing global efforts to control or eliminate human helminthiases through mass drug administration (MDA), vector control, and the delivery of new-generation biotechnologies. Because worms exist in dynamic relationships with their environments and, for some, with snail intermediate hosts or insect vectors, the effects of climate change will vary by species and could be multidimensional in nature. In addition, climate change may act synergistically or competitively with the major social determinants highlighted above, as well as MDA.

Schistosomiasis in Africa is an example of the complexities of climate change interacting with urbanization and MDA. The hotter and drier conditions expected for many parts of Africa might reduce the continent’s overall incidence of schistosome infections by decimating freshwater snail populations, which serve as intermediate hosts for the digenetic blood trematodes [3]. However, not all species will suffer ill effects of environmental change, and these differential responses will influence disease burden. Ultimately, Africa’s anticipated future economic development—together with expansion of praziquantel MDA and the possible development of a schistosomiasis vaccine—might eventually reinforce the effects of snail decimation due to climate change, thereby accelerating the elimination of schistosomiasis on the African continent.

In contrast to the example above, in the case of lymphatic filariasis (LF), which has been slated for elimination via MDA [4], expanded ranges and extended breeding seasons of mosquitos (the disease’s transmitting vector) [5,6] could make that goal unattainable. For LF, global elimination efforts may depend on a race between MDA and climate change-induced expansion of vector ranges, or they might require introducing new or intensified vector control management programs.

Climate change could also upset the dominance of certain worm species within a related group of parasites. Whereas *Necator americanus* is currently the predominant hookworm worldwide [7], changing patterns of drought or temperature might ultimately favor *Ancylostoma duodenale* due to its unique ability to undergo developmental arrest as dauer larvae in human tissues as a means to survive environmental extremes [7,8].

Predicting clearly defined outcomes of climate change can be exceedingly complex. This is because most research suggests that hotter temperatures and altered rainfall can favor or disfavor some species of helminths or their snail (or insect-vector) hosts over others. It is also possible that hotter temperatures and droughts can work synergistically or in competition with key social determinants and control efforts through MDA. Furthermore, infrastructural developments and subsequent changes in human exposure add another dimension to consider, especially in parts of Africa and China that are rapidly altering local ecosystems through construction of dams and other projects. Ultimately, climate change forecasts can be used to guide and inform future treatment plans. Described below are some possible global trends for the major worm species currently infecting large human populations.

## Human soil-transmitted helminthiases: Ascariasis, Trichuriasis, and hookworm infections

Differences in the fundamental biology and thermotolerance of the three major soil-transmitted helminths could play a large role in determining each one’s prevalence in the coming decades. In Africa, some investigators consider hookworms the best suited to adapt to climate change by virtue of several key characteristics. First, hookworm larvae in African soils are reported to remain viable up to a land surface temperature (LST) of 40°C or higher [9–11], unlike *Ascaris lumbricoides* and *Trichuris trichiura* eggs that cease to develop at 38°C [8,10]. Further, the motility of infective hookworm larvae [8,10,12] and accelerated development (3–10 days versus 10–30 days and 28–84 days for *T*. *trichiura* and *A*. *lumbricoides* eggs, respectively) could confer advantages, as do adult hookworms’ longer lifespans [8]. The finding that hookworm is seldom found in areas where the mean LST falls below 10°C during the warmest quarter of the year means that warming may increase the geographic range of hookworm to the southernmost areas of Africa [9]. Currently in Africa, both *N*. *americanus* and *A*. *duodenale* are present, although the former dominates in terms of overall prevalence. With greater environmental extremes, *A*. *duodenale* might begin outcompeting *N*. *americanus* through its ability to undergo arrested development in human tissues and remain shielded from the outside climate. Thus, *A*. *duodenale* could become a dominant soil-transmitted helminth on the African continent.

However, studies of the same four nematode species in Asia suggest potentially different outcomes such that *A*. *lumbricoides* may become dominant in that region by virtue of its ability to more successfully endure hotter temperatures and higher aridity, such as those found in India or Pakistan [8,9,11]. As Pullan and Brooker point out, it remains unclear why hotter soil temperatures in Africa favor hookworm larvae, but hotter temperatures in Asia do not similarly diminish *Ascaris* egg development. In addition, because *A*. *lumbricoides* can survive in urban environments, we might expect to see continued high rates of human ascariasis in Asian megacities [13].

Importantly, before reaching soil-transmitted helminth egg and larval upper LST development thresholds, warmer temperatures generally increase the rates of parasite development [11]. These findings suggest that mild climate change-related increases in temperature could facilitate higher rates of infectivity and heavier burdens of disease in the immediate future before becoming a limiting factor in 2030–2040.

## Human trematodiases: Schistosomiasis and liver fluke infection (opisthorchiasis and fascioliasis)

For parasitic worms that have digenetic lifecycles or otherwise require secondary hosts in their reproductive cycles, determining the effects of climate change demands analyses of how snail intermediate hosts will fare in the coming decades. In the case of schistosomiasis, some studies suggest that continuous high temperatures in parts of Africa, such as coastal East Africa and elsewhere, explain why *Biomphalaria* spp. cannot survive or sustain *Schistosoma mansoni* transmission [14]. Therefore, several studies of various host snail species in Africa indicate that (with some exceptions) climate change linked to higher temperatures, as well as periods of drought or algal blooms, could significantly reduce the range and abundance of these intermediate hosts [14–16]. However, habitat contractions in some parts of Africa might be partially compensated by schistosome-transmitting snails entering cooler areas of Southern Africa [14,16]. Similarly, global warming could potentially promote the expansion of *Oncomelania* snails transmitting *Schistosoma japonicum* into new Asian niches [14].

Beyond the detrimental changes affecting snail aquatic environments such as those highlighted above, schistosome snail intermediate hosts may also face higher mortality rates associated with increased cercarial production at elevated temperatures [14,17]. In addition, higher temperatures may decrease cercarial survival [14]. Taken together, these factors have led some to predict a 13%–19% contraction in transmission area of *S*. *mansoni* infection in Africa by 2080 [16]. In East Africa, however, climate change could cause range shifts without substantially affecting increases or decreases in overall disease burden [18].

Liver fluke infection is a significant cause of disability-adjusted life years and bile duct cancers in Asia and Russia. Significant environmental factors determining the distributions of *Opisthorchis viverrini* and its snail intermediate hosts in Thailand include precipitation and minimum temperature, such that transmission might increase in the immediate future, but overall, snail habitats will contract by 2050 and continue to shrink through 2070 [19]. Additionally, ongoing construction of dams on the Mekong River will affect fisheries and, in turn, *O*. *viverrini* transmission in regional locales. Potentially, similar restrictions on habitats for snails that transmit *Clonorchis sinensis* in China and Korea or *Opisthorchis felineus* in Kazakhstan and Russia will also result in overall lower global prevalence rates of human liver fluke infections. For human fascioliasis, the periods of drought and water stress in Egypt may also cause altered or limiting transmission patterns [14].

## Insect-borne helminthiases: LF and onchocerciasis

WHO has identified LF as a target for elimination by 2020 and is working toward that goal through expansion of MDA in the affected countries, but the effects of climate change could interfere with these efforts.

There are several species of filarial-transmitting mosquitos that could proliferate through future expansions of tropical and subtropical regions [5, 11]. Indeed, one study used maximum entropy ecological niche modeling to predict that the current at-risk population could grow from 543–804 million (a figure approximately in line with current WHO estimates) to 1.65–1.86 billion due to the effects of climate change and population growth [20]. Further, a 2007 surveillance study of vector-borne diseases in Nepal found that there has been a shift of LF transmission from lowland and hill districts (targeted by MDA campaigns beginning in 2003) to the country’s mountain regions [6]. Areas of higher altitude were previously considered low-risk due to the inability of mosquito vectors to survive there. Thus, anticipated climate changes might be advantageous for LF transmission and could demand an expansion in global MDA or vector control efforts, especially in areas that were not previously considered vulnerable for LF endemicity.

Similarly, climate change appears to favor higher transmission rates of onchocerciasis by virtue of faster parasite development rates and expanded blackfly vector ranges for selected *Simulium* species [21]. A study of *Onchocerca volvulus* in Liberia and Ghana found that maximally favorable blackfly conditions would occur at temperatures 3°C and 7°C above current monthly averages in each country, respectively, but could decline thereafter [21]. However, competing with these effects are possible reductions in selected blackfly populations attributable to findings that different *Simulium* species thrive best at different maximal temperatures. For example, savannah-adapted sandflies, which thrive best at higher temperatures, may replace forest-adapted species as global warming progresses [21]. In this race against global warming, there may be a need to accelerate onchocerciasis elimination efforts by prioritizing the development of onchocerciasis preventative vaccines [22].

## Conclusion

The most dramatic effects of climate change—such as rising sea levels, floods and droughts, loss of biodiversity, and extreme weather events—pose significant threats to planetary health by the end of the century. With regards to human infection, we expect that hookworm will remain a major soil-transmitted helminth infection but that ancylostomiasis in particular might emerge as a new dominant soil-transmitted helminth infection in Africa, with ascariasis predominating in Asia (Box 1). A predicted demise of intermediate snail hosts could lead to decreases in the prevalence of schistosomiasis in traditionally endemic areas of Africa. Similarly, contractions in habitats suitable for transmission of opisthorchiasis and clonorchiasis could reduce the burden of liver fluke disease in East Asia, although moderate increases in temperature could temporarily lead to an increased prevalence in the immediate future. In contrast, climate change might favor the transmission of LF and onchocerciasis by expanding the ranges of mosquitos and blackflies, respectively.

**Box 1. Summary of the major effects of climate change on human helminth infections**

- Soil-transmitted helminth infections
  ○ Hookworm (especially ancylostomiasis) emerges as a dominant infection in Africa.
  ○ Ascariasis remains the dominant infection in warming Asian megacities.
- Trematodiases
  ○ Decline of schistosomiasis in Africa due to shrinking snail host habitats, droughts, accelerated cercarial development, and human intervention—alternative scenario of snails expanding their habitat by moving into cooler areas.
  ○ Likely decline of opisthorchiasis in Asia.
- Filarial nematode infections
  ○ Predicted expansion in range and breeding season for mosquito insect vectors.
  ○ Race between global warming and sustainable MDA for LF and onchocerciasis.

As has been duly noted by a variety of concerned stakeholders, including the United Nations [23], former Vice President Al Gore, and the Vatican [24], the effects of climate change will disproportionately affect the poorest of the poor. It is therefore crucial that policy makers and organizations aiming to improve human health leverage the information currently available and consider accelerating global elimination efforts of NTDs as a means to reduce suffering among populations whose multifaceted impoverishment will be exacerbated by climate change in the coming decades.
